# Supplementary material for: Evaluation of unmanned aerial vehicle shape, flight path and camera type for waterfowl surveys: disturbance effects and species recognition
Source: PeerJ. 2016 Mar 21;4:e1831. doi: 10.7717/peerj.1831 (PMC4806640; doi:10.7717/peerj.1831)
Supplement: Supplemental Information 1 — List of bird species observed at two study sites, Little Llangothlin Lagoon and Lake Cargelligo sewage works, NSW between March and May 2015. [file peerj-04-1831-s001.docx]

# List of bird species observed at each study site

| Little Llangothlin Lagoon, NSW March-May 2015 | |
| --- | --- |
|  |  |
|  |  |
|  |  |
| **Common Name** | **Scientific Name** |
| **Water Birds** |  |
| Grey Teal | Anas gracilis |
| Pacific Black Duck | Anas superciliosa |
| Australasian Shoveller | Anas rhynchotis |
| Australian Wood Duck | Chenonetta jubata |
| Blue Billed Duck | Oxyura australis |
| Freckled Duck | Stictonetta naevosa |
| Pink Eared Duck | Malacorhynchus membranaceus |
| Hard Head | Aythya australis |
| Musk Duck | Biziura lobata |
| Eurasian Coot | Fulica atra |
| Black Swan | Cygnus atratus |
| Australasian Grebe | Tachybaptus novaehollandiae |
| Hoary Headed Grebe | Poliocephalus poliocephalus |
| Yellow Billed Spoonbill | Plataelea flavipes |
| Royal Spoonbill | Platalea regia |
| Great Cormorant | Phalacrocorax carbo |
| Straw Necked Ibis | Threskiornis spinicollis |
| Black Winged Stilt | Himantopus leucocephalus |
| Red Necked Avocet | Recurvirostra novaehollandiae |
| Red Kneed Dotterel | Erythrogonys cinctus |
| Black Fronted Dotterel | Elseyornis melanops |
| Masked Lapwing | Vanellus miles |
| Intermediate Egret | Ardea intermedia |
| White Faced Heron | Egretta novaehollandiae |
| Australian Pelican | Pelecanus conspicillatus |
| White Necked / Pacific Heron | Ardea pacifica |
| **Raptors and Corvids** |  |
| Australian Raven | Corvus coronoides |
| Brown Falcon | Falco berigora |
| Australian Hobby | Falco longipennis |
| Black Kite | Milvus migrans |
| Whistling Kite | haliastur sphenurus |
| Marsh Harrier | Circus approximans |
| White Bellied Sea Eagle | Haliaeetus leucogaster |
| **Parrots** |  |
| Galah | Eolophus rosiecapillus |
| Crimson Rosella | Platycercus elgans |
| **Passerines** |  |
| Australian Magpie | Cracticus tibicen |
| Red Capped Robin | Petroica goodenovii |
| Magpie Lark | Grallina cyanoleuca |
| Tree Martin | Petrocheildon nigricans |
| Willie Wagtail | Rhipidura leucophrys |

| Lake Cargelligo Bird List March -May 2015 | |
| --- | --- |
|  |  |
|  |  |
| **Common Name** | **Scientific Name** |
| **Water Birds** |  |
| Grey Teal | Anas gracilis |
| Pacific Black Duck | Anas superciliosa |
| Australian Wood Duck | Chenonetta jubata |
| Pink Eared Duck | Malacorhynchus membranaceus |
| Hard Head | Aythya australis |
| Eurasian Coot | Fulica atra |
| Purple Swamphen | Porphyrio porphyrio |
| Black Swan | Cygnus atratus |
| Hoary Headed Grebe | Poliocephalus poliocephalus |
| Australasian Grebe | Tachybaptus novaehollandiae |
| Black Tailed Native Hen | Tribonyx ventralis |
| Little Pied Cormorant | Microcarbo melanoleucos |
| Black Winged Stilt | Himantopus leucocephalus |
| Australian Pelican | Pelecanus conspicillatus |
| Australasian Darter | Anhinga novaehollandiae |
| Red Kneed Dotterel | Erythrogonys cinctus |
| Black Fronted Dotterel | Elseyornis melanops |
| White Necked / Pacific Heron | Ardea pacifica |
| **Raptors and Corvids** |  |
| Australian Raven | Corvus coronoides |
| Nankeen Kestrel | Falco cenchroides |
| Brown Falcon | Falco berigora |
| Black Kite | Milvus migrans |
| Whistling Kite | haliastur sphenurus |
| White Bellied Sea Eagle | Haliaeetus leucogaster |
| **Parrots** |  |
| Red Rumped parrot | Psephotus haematonotus |
| Mallee Ringneck | Barnardius zonarius barnardi |
| Cockatiel | Nymphicus hollandicus |
| Galah | Eolophus rosiecapillus |
| Major Mitchell's Cockatoo | Lophocroa leadbeateri |
| **Passerines** |  |
| Australian Magpie | Cracticus tibicen |
| Crested Pigeon | Ocyphaps lophotes |
| White Fronted Chat | Epthianura albifrons |
| Red Capped Robin | Petroica goodenovii |
| White Plumed Honeyeater | Lichenstomus penicillatus |
| Singing Honeyeater | Lichenostomus virescens |
| Yellow Throated Miner | Manorina melanotis |
| Magpie Lark | Grallina cyanoleuca |
| Richard's Pipit | Anthus novaeseelandiae |
| Variegated Fairy Wren | Malurus lamberti |
| Tree Martin | Petrocheildon nigricans |
| Pied Butcher Bird | Cracticus nigrogularis |
| Grey Butcher Bird | Craticus torquatus |
| Black Faced Cuckoo Shrike | Coracina novaehollandiae |
